# Use of cidofovir in pediatric patients with adenovirus infection

**DOI:** 10.12688/f1000research.8374.2

**Published:** 2016-12-16

**Authors:** Lakshmi Ganapathi, Alana Arnold, Sarah Jones, Al Patterson, Dionne Graham, Marvin Harper, Ofer Levy

**Affiliations:** 1 Division of Infectious Diseases, Boston Children’s Hospital, Boston, MA, USA; 2 Harvard Medical School, Boston, MA, USA; 3 Division of Pharmacy, Boston Children’s Hospital, Boston, MA, USA; 4 Program for Patient Quality and Safety, Boston Children’s Hospital, Boston, MA, USA

**Keywords:** cidofovir, anti-viral, pediatric, adenovirus, stem-cell, solid organ, immunocompromised

## Abstract

**Background**: Adenoviruses contribute to morbidity and mortality among immunocompromised pediatric patients including stem cell and solid organ transplant recipients. Cidofovir (CDV), an antiviral compound approved by the FDA in 1996, is used for treatment of adenoviral (ADV) infections in immunocompromised patients despite concern of potential nephrotoxicity.

**Methods**: We conducted a retrospective 5-year review at Boston Children’s Hospital of 16 patients (mean age = 6.5 years) receiving 19 courses of CDV. During therapy all pertinent data elements were reviewed to characterize potential response to therapy and incidence of renal dysfunction.

**Results:** Of the 19 CDV courses prescribed, 16 courses (84%) were in patients who had a positive blood ADV Polymerase chain reaction (PCR) alone or in combination with positive ADV PCR/ Direct Immunofluorescence Assay (DFA) at another site. Respiratory symptoms with or without pneumonia were the most common presentation (10/19, 53%). In the majority of blood positive courses (10/16, 63%), viral clearance was also accompanied by clinical response. This was not the case in four courses where patients expired despite viral clearance, including one in which death was directly attributable to adenovirus. There was reversible renal dysfunction observed during the use of CDV.

**Conclusions:**  CDV appeared safe and reasonably tolerated for treatment of ADV in this pediatric population and was associated with viral response and clinical improvement in the majority of patients but reversible renal dysfunction was a side effect. Further studies of the efficacy of CDV for immunocompromised children with ADV infection are warranted.

## Introduction

Adenovirus (ADV) is a common cause of respiratory infection in childhood. ADV infections are usually self-limited and asymptomatic in the immunocompetent host but have been recognized as a cause of significant morbidity and mortality in immunocompromised pediatric patients such as recipients of hematopoietic stem cell transplant (HSCT) and solid organ transplant (SOT) ^1^. In these patients, ADV is an opportunistic pathogen that may lead to severe localized disease including pneumonia/pneumonitis, hepatitis, hemorrhagic cystitis or disseminated disease with multiorgan failure ^2– 4^. Case fatality rates in immunocompromised patients with ADV pneumonia have been reported to be as high as 60% ^5^. Currently, there is no FDA-labeled product available for treatment of ADV infection though several agents have been administered for this indication including ribavirin ^6, 7^, ganciclovir ^8^, vidarabine ^9, 10^, immune globulin ^11^ and cidofovir ^12– 21^.

Cidofovir (CDV), a nucleoside and phosphonate analogue is a broad-spectrum antiviral agent that inhibits viral DNA polymerase and has broad activity *in vitro* against multiple viruses including all serotypes of ADV ^22, 23^. CDV has an FDA indication for the treatment of cytomegalovirus (CMV) retinitis in patients with AIDS. Although this drug does not have an FDA indication for treating ADV, there is evidence of *in vivo* efficacy of CDV against ADV ^12, 14^. While CDV at a standard dose of 5mg/kg has been reported as primary therapy for treatment of ADV infection in pediatric and adult hematopoietic stem cell transplantation (HSCT) patients ^12, 21^, concern exists regarding potential nephrotoxicity. This associated adverse effect has limited the use of CDV for treatment of ADV infections in pediatric patients. To minimize potential toxicity of CDV, modified dosing regimens such as the use of 1 mg/kg three times weekly have been utilized ^14^.

Limited information regarding safety and efficacy of CDV in pediatric patients prompted us to review prior published studies in the literature and conduct a retrospective review of our inpatient use of CDV at Boston Children’s Hospital (BCH).

## Methods

Following Institutional Review Board (IRB) approval (IRB-P00015576), a retrospective chart review was conducted for all hospitalized patients at Boston Children’s Hospital (BCH), who were prescribed CDV for adenovirus infection from January 2006 through December 2010. Depending on the clinical context, testing for adenoviruses was typically only performed in symptomatic patients, except in the case of stem cell transplant recipients who had a known history of adenovirus infection prior to transplant in which case adenovirus screening was performed routinely. The following data were collected: (1) demographic information, (2) underlying disease state, (3) type of transplant, (4) duration of cidofovir therapy, (6) serum creatinine (SCr) (baseline, peak during therapy, and level up to 2 weeks post last dose), (7) concomitant nephrotoxins prescribed (acyclovir, amikacin, cyclosporine, foscarnet, gentamicin, liposomal amphotericin B, tacrolimus, tobramycin, vancomycin, and intravenous contrast media), (8) sites of ADV detection by viral direct fluorescent antibody (DFA), nucleic acid test, and/or culture, (9) viral quantitative PCR surveillance in whole blood and other sites of infection (all specimens were tested at least weekly before, during and to two weeks post last dose of CDV to evaluate for changes in viral load with a minimum three serial values being obtained before, during and at end of therapy); (10) symptoms of infection, and clinical course including response to therapy, (11) concomitant reduction of immunosuppression and (12) mortality and cause(s) of mortality. All blood sample testing for adenovirus quantitative PCR in whole blood was performed at the Boston Children’s Hospital Virology Laboratory using our laboratory developed test, using components of the Argene adenovirus kit (bioMerieux, Cambridge, MA). The Argene adenovirus kit contains primers and probes selective for a 138 base pair (bp) sequence in the Hexon gene of the adenovirus. Using a 5’ nuclease assay, viral DNA is detected using the primers and fluorescent probes from the Argene kit by means of real time PCR in a Cepheid SmartCycler (Cepheid, Sunnyvale, CA). Of note, all patients who received cidofovir also received probenecid for renal protection at a standard dose of 1.25g/m ^2^/dose administered 3 hours prior to and 2 hours and 8 hours after completion of each 1-hour CDV infusion. We performed a search of relevant literature up till December 2010. The literature search was conducted using PubMed, PubMed Central and Medline databases. Search terms included “treatment of adenoviral infection”, “pediatric”, “adenovirus” “lung transplant”, “liver transplant”, “multi-visceral transplant”, “kidney transplant”, “stem cell transplant”, “immunocompromised”, “nephrotoxic”, “ribavirin”, “ganciclovir” and “vidarabine”. As we found larger case series pertaining to use of cidofovir in stem cell transplant recipients, we excluded case reports in this population. However, given the paucity of data in solid organ transplant recipients, we included case reports.

### Definitions

As there is no accepted definition for ADV infection or disease, we adopted definitions used in prior studies ^13^. Specifically, *definite adenovirus disease* as follows: *Non-gastrointestinal locations*: Symptoms and signs from the appropriate organ combined with histopathological documentation of adenovirus and/or adenovirus detection by culture, antigen test, or nucleic acid test from biopsy specimens (liver or lung), BAL fluid, or cerebrospinal fluid and without another identifiable cause; *Gastrointestinal location*: Symptoms together with detection of adenovirus from biopsy material by culture, antigen test, or nucleic acid test.

*Probable adenovirus disease* as *follows: Gastrointestinal tract*: Detection of adenovirus in stool by culture, antigen test, or nucleic acid test together with symptoms; *Urinary tract*: Symptoms of dysuria or hematuria combined with detection of adenovirus by culture, antigen test, or nucleic acid test without another identifiable cause; and *Respiratory tract*: Symptoms and signs of pneumonia/pneumonitis combined with detection of adenovirus by culture, antigen test, or nucleic acid test without another identifiable cause.

*Asymptomatic adenovirus infection* as follows: any detection of adenovirus in an asymptomatic patient from stool, blood, urine, or upper airway specimens by viral culture, antigen tests, or PCR.

*Adenoviremia* was defined as the detection of >100 copies of ADV/mL of blood (this being the lower limit of detection of the assay). Viral clearance was defined as an ADV viral load of <100 copies in blood by quantitative PCR at the end of therapy. Viral response was defined as decrease in viremia by at least one log-reduction (i.e 10-fold). Clinical resolution was defined as resolution of symptoms and/or signs of infection.

*Renal dysfunction* was defined as a ≥50% increase in SCr from baseline during the course of CDV therapy. Baseline creatinine was defined as the most recent serum creatinine value prior to initiation of CDV therapy.

### Statistical analysis

Statistical analyses employed Prism 5 for Windows Version 5.04 (GraphPad Software Inc, CA). The Mann-Whitney test was used to assess risk of renal dysfunction. Trends in adenoviremia including pre-treatment viral load, changes in viral load during therapy, and post-treatment viral load were graphed.

## Results

**Figure 1.**
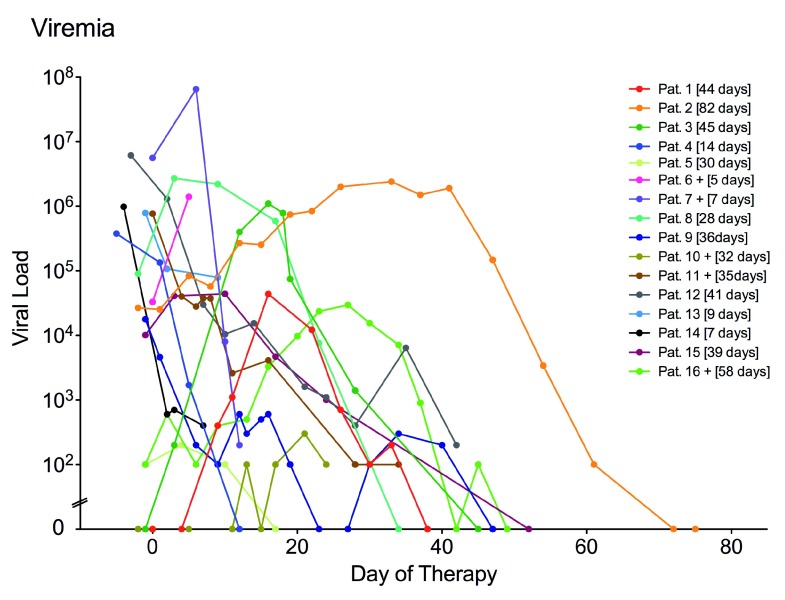
Kinetics of Adenovirus blood viral load under cidofovir treatment. Viral loads of 16 patients with quantitative blood adenovirus PCR treated with cidofovir, are shown. Day of therapy ≤0 denotes pre-treatment viral loads. Up to two post-treatment values are shown where available and informative. Each patient’s individual treatment duration is shown in the legend. + denotes patient expired.

Raw data for Figure 1 Adenovirus blood viral load is represented in each column at each particular time in days (before or after onset of cidofovir treatment) for each patient. Click here for additional data file. Copyright: © 2016 Ganapathi L et al. 2016 Data associated with the article are available under the terms of the Creative Commons Zero "No rights reserved" data waiver (CC0 1.0 Public domain dedication).

Raw data for Figure 2 Nephrotoxicity - pre, peak and post serum creatinine levels in mg/dL are represented in each column for each patient. Click here for additional data file. Copyright: © 2016 Ganapathi L et al. 2016 Data associated with the article are available under the terms of the Creative Commons Zero "No rights reserved" data waiver (CC0 1.0 Public domain dedication).

From January 1, 2006 to December 31, 2010, a total of 16 pediatric patients received CDV for adenovirus infection at our hospital. These 16 patients received 19 courses (three patients received two separate CDV courses). The standard CDV dose of 5mg/kg weekly was used in all courses unless there was concern for renal dysfunction at the start of therapy in which case a dosing regimen of 1mg/kg three times a week was used. Patient demographics, primary diagnosis, clinical symptoms and course, and sites of adenovirus detection appear in Table 1. Patient age ranged from 0.75–20 years (mean 6.5 years). Seven (44%) patients were male. Underlying primary diagnosis included 8 (50%) HSCT (1 autologous), 4 (25%) SOT, 2 (12.5%) leukemia, and 2 (6.5%) defined as other. Duration of CDV therapy ranged from 5–82 days (median 33.5 days).

**Table 1.** Demographics, primary diagnosis, sites of ADV detection, clinical symptoms and course of patients included in the study. The age and gender distribution, primary diagnosis, sites of adenovirus detection, symptoms and clinical course of the patients included in the study are shown.

Pt #	Age (yrs)	Gender	Diagnosis	Site(s) of ADV detection	Clinical symptoms
1a	12	F	AML - Mismatched UD Cord SCT	Sp, S, R	Pneumonia
1b	12	F	AML - Mismatched UD Cord SCT	Sp	Fever and Respiratory symptoms
2	12	M	Severe Idiosyncratic Immunodeficiency - MRD SCT	B, S	Prolonged fever
3	3	M	AML - Chemotherapy	B, S, U	Pneumonia
4	4	F	Neuroblastoma - Autologous SCT	B, S, PF	Prolonged fever, pericardial effusion
5	19	F	Cystic Fibrosis - Lung Transplant	B, BAL	Asymptomatic
*6	1	F	Familial HLH - MUD SCT	B, Sp	Pneumonia, sepsis. Other co-infections including Enterococcus bacteremia, EBV viremia
*7	0.83	M	Persistent pulmonary hypertension, cardiomyopathy	B	Fever, sepsis, pneumonia. Other co-infections including stenotrophomonas bacteremia
8a	20	M	ALL - MRD SCT and MUD SCT	BAL, B, U	Pneumonia
8b	20	M	ALL - MRD SCT and MUD SCT	S	Asymptomatic
9	1	F	Congenital Nephrotic Syndrome & Hepatoblastoma - Combined Liver & Kidney Transplant	B,Sp	Respiratory symptoms
*10	0.75	M	HLH - SCT	S, B, U	Fever, sepsis, diarrhea
*11	5	F	CID and Lymphoproliferative Disorder - Mismatched UD Cord SCT	B	Fever, respiratory symptoms, diarrhea, hemorrhagic cystitis
12	2	F	Congenital Cardiac Defect - Heart Transplant	B	Fever, sepsis
13	2	F	ALL - Chemotherapy	B, S	Fever, respiratory symptoms
14	2	F	Cerebral Palsy	B	Pneumonia
15a	1.8	M	Hepatoblastoma - Multivisceral Transplant	B, S	Increased stoma output, rejection, biopsy proven ADV In stoma mucosa
15b	1.8	M	Hepatoblastoma - Multiviseral Transplant	B, S	Increased stoma output
*16	17	M	AML - MUD SCT	B, U	Hemorrhagic cystitis. Other co-infections including BK viruria and EBV viremia

ALL, acute lymphoblastic leukemia; AML, acute myeloid leukemia; CID, congenital immunodeficiency; CML, chronic myelogenous leukemia; HLH, hemophagocytic lymphohistiocytosis; MRD, matched related donor; MUD, matched unrelated donor; Pt #, patient number; SCID, severe combined immunodeficiency disorder; SCT, stem cell transplant; UD, unrelated donor; Yrs, years; Site of adenovirus detection: S, stool, Sp, sputum, B, blood, BAL, bronchoalveolar lavage, R, respiratory DFA, CSF, cerebrospinal fluid, U, Urine, PF, Pericardial Fluid; *indicates patient expired

Of the 19 courses prescribed ( Table 1), two courses were prescribed in a patient with definite adenovirus disease of the gastrointestinal tract, 15 courses were prescribed in patients with probable disease and two courses were prescribed in patients with asymptomatic infection. Sixteen courses (84%) were in patients who had a positive blood ADV PCR either in whole blood only or in combination with positive ADV PCR of sputum, stool, urine, broncho-alveolar lavage (BAL) fluid, pericardial fluid or positive sputum adenoviral DFA sample. Respiratory symptoms were the most common presentation (10 courses, 53%) of which six courses were prescribed for patients with respiratory symptoms and radiological evidence of pneumonia. Two courses were prescribed in patients who presented with prolonged fevers; four courses were prescribed in patients who had worsening diarrhea and colitis, two of which were biopsy proven adenovirus infection; four courses were prescribed in patients with viral sepsis with or without pneumonia; and two courses were administered in patients with severe hemorrhagic cystitis. One course was prescribed in a patient with asymptomatic respiratory tract infection and one course was prescribed in another patient with asymptomatic gastrointestinal infection.

We further examined the 16 blood-positive courses to assess trends in ADV viral load pre-, during and post- CDV therapy ( Figure 1). A quantitative reduction in viral load was seen in 15 blood positive courses (94%) with viral clearance achieved in 14 (88%). Of note, all solid organ transplant recipients treated with CDV also had concomitant decrease in their immunosuppression. A single patient (Patient 6) did not demonstrate viral response to therapy and expired. The majority of adenovirus blood-positive CDV courses (10/16, 63%) were associated with clinical improvement with viral clearance; however this was not the case in four courses. Patients 7, 10, 11 and 16 expired despite demonstrating viral clearance. Patients 6, 7 and 16 had multiple other co-infections. Patients 11 and 16 developed severe hemorrhagic cystitis. Patient 11 experienced significant complications of hemorrhagic cystitis including urinary tract obstruction, renal failure and bladder perforation. Patient 16 also had concomitant BK Polyoma virus detected in the urine.

Each patient’s medication profile was assessed to determine the number of additional nephrotoxic agents concomitantly prescribed during CDV therapy (from Day 1 to 7 days post last CDV dose) ( Table 2).

**Table 2.** Additional nephrotoxic agents prescribed and changes in serum creatinine during cidofovir therapy. Absolute values for serum creatinine in mg/dL for each patient are represented pre-treatment, during treatment (peak serum creatinine), and post-treatment. Additional nephrotoxic agents that each patient received are also represented.

Pt #	Pre-treatment Serum Cr	Peak Serum Cr	Post-treatment Serum Cr	Additional nephrotoxic agents
1a	0.3	1.2	0.6	ambisome, cyclosporine, gentamicin
1b	0.6	1	0.6	cyclosporine
2	0.6	0.6	0.5	cyclosporine, ganciclovir, pentamidine
3	0.3	0.6	0.2	vancomycin, ambisome, gentamicin
4	0.3	0.3	0.2	acyclovir
5	0.4	0.7	0.5	tacrolimus
6	0.2	0.4	0.6	ambisome, ganciclovir, pentamidine, tacrolimus, vancomycin
7	0.2	0.5	0.7	gentamicin,
8a	1	1.7	0.4	ambisome, vancomycin
8b	0.4	2.6	1.3	ganciclovir, tacrolimus, vancomycin
9	0.3	2.7	0.6	chemotherapy, immunosuppression
10	0.2	0.2	0.2	acyclovir, ambisome, cyclosporine, vancomycin
11	0.4	1.6	0.6	gentamicin, acyclovir, ambisome, cyclosporine, foscarnet, ganciclovir
12	0.5	1.6	0.6	gentamicin, ganciclovir, tacrolimus
13	0.2	0.2	0.2	ambisome,foscarnet,ganciclovir
14	0.2	0.2	0.2	vancomycin
15a	0.3	0.3	0.3	tacrolimus, vancomycin
15b	0.2	0.3	0.2	tacrolimus, vancomycin
16	0.3	0.5	0.3	cyclosporine, gentamicin, vancomycin, contrast

Pt #, patient number; Cr, creatinine; all creatinine values are in mg/dL.

Administration of CDV was significantly associated with occurrence of renal dysfunction when comparing the peak Cr measured during CDV therapy to the baseline serum Cr (p=0.0016). Eleven courses (58%) were associated with development of renal dysfunction. Cr increased by a mean of ~50% from baseline during CDV therapy.. Of the courses with elevation in serum creatinine, 64% demonstrated return to pre-treatment creatinine levels following cessation of CDV therapy.

## Discussion

In this retrospective review of patients treated with CDV for adenovirus infection at our hospital during a 5-year period, we assessed the safety and potential efficacy of the medication in pediatric patients. Our review yielded a case series of 16 patients. While the number of patients is modest, this series adds to the existing literature describing the use of CDV in pediatric recipients of HSCT, SOT and chemotherapy for oncologic diagnoses ( Table 3).

**Table 3.** Summary of studies describing use of cidofovir in pediatric HSCT and SOT recipients. Published studies in the literature describing use of cidofovir in pediatric HSCT and SOT recipients are summarized. Toxicities, and clinical outcomes reported in each study are highlighted.

Study	# Patients (N)	Clinical Setting	Median Age	Cidofovir dosage	Duration of cidofovir use	Potential Toxicity	Outcome
Hoffman ^14^	8	HSCT	7 yrs	1 mg/kg/dose three times weekly x 9 doses or until clearance	3 weeks-8 months	Well tolerated; no toxicity reported	100% viral suppression 3 recurrences 4 expired (2 ADV-related)
Muller ^12^	10	HSCT	Not reported	5 mg/kg/dose weekly up to 6 weeks, then every other week for 3 more doses	3 weeks-6 months	30% nephrotoxicity (50% increase Cr)	9 virologic clearance 5 recurrences 1 expired (interstitial pneumonitis)
Anderson ^19^	7	HSCT	1.5 yrs	Preemptive therapy: 1 mg/kg/ dose three times weekly x 3 weeks	3 weeks	Well tolerated without significant toxicity reported	No patient developed ADV viremia 2 expired (non- ADV related)
Bhadri ^15^	23	87% HSCT 13% oncologic receiving chemotherapy	5.7 yrs	5 mg/kg/dose weekly, 3 mg/kg/ dose weekly or 1 mg/kg/dose three times weekly	Median 6 weeks (1–26 weeks)	9% Grade 1 nephrotoxicity defined by increase of creatinine up to 1.5 times upper limit of normal	85% of 20 evaluable patients considered successful by Ljungman criteria ^13^ 17 expired
Yusuf ^17^	57	90% HSCT 10% oncologic receiving chemotherapy	8 yrs	5 mg/kg/dose weekly x2 weeks, then every other week until 3 negative ADV samples	Median 60 days (1 week-9 months)	No toxicity reported	98% successful viral clearance 14% recurrence 29 expired (1 ADV-related death)
Legrand ^18^	7	HSCT	6.4 yrs	5 mg/kg/dose weekly x3 wks then every other wk or 10 days	25–330 days	43% nephrotoxicity	71% deemed recovered 2 expired (1 ADV related death)
Sivaprakasam ^26^	8	HSCT	11 yrs	1 mg/kg/dose 3 times weekly (4 patients also received IV ribavirin 5 mg/kg 3 times daily)	Not reported	2 cases marrow failure, 1 case nephropathy	3 expired (attributed to ADV and GVHD)
Williams ^27^	9	HSCT	3 yrs	5 mg/kg/dose once weekly until 3 weeks of negative results or pt no longer high risk; if underlying renal dysfunction 1 mg/kg/dose 3 times weekly	Median 8 doses (3–32 doses)	22% renal failure (compared to 80% untreated comparator group)	89% ADV clearance 3 expired (1 ADV related)
Engelmann ^16^	1	Liver transplant	7 months	6 mg/kg/dose x1 with 1 repeat dose 6 days later	2 weeks	No toxicity reported	Liver rejection; reported to have slow recovery
Wallot ^20^	2	Liver transplant	8 months and 14 months	1 mg/kg/dose three times weekly	5–8 weeks	1 moderate neutropenia, 1 transient rise in creatinine	Blood PCR ADV clearance in both patients No deaths
Carter ^24^	1	Liver transplant	7 months	1 mg/kg/dose three times weekly	7 weeks	Transient acidosis and proteinuria	ADV viral culture and blood PCR became negative
Doan ^25^	4	Lung transplant	<3 yrs	1 mg/kg/dose every other day to three times weekly plus IVIG (1 pt increased dose to 2 mg/kg/ dose; 1 pt increased frequency to daily therapy x 2 weeks)	4 weeks	No toxicity reported	75% negative blood ADV PCR 1 death
Siedemann ^29^	6	70% Liver transplant 15% HSCT 15% combined liver and HSCT	2 yrs	5 mg/kg/dose once weekly	Not reported	Not reported	3 expired (all ADV related)
Leurez-Ville ^30^	37 (8 patients with disseminated infection)	60% HSCT 20% solid organ transplant (1 liver, 2 small bowel, 5 combined liver and small bowel) 20% congenital immunodeficiency	2.2 yrs (only cases of disseminated infection reported)	5mg/kg/dose once weekly for two weeks followed by 5mg/kg every 2 weeks or 10 days; only patients with disseminated infection received cidofovir)	Not reported	1 patient with persistent nephroxicity requiring dose adjustment of cidofovir	60% with disseminated infection recovered 3 patients with disseminated infection expired

Similar to other studies the majority of our patients had received a HSCT or had an oncologic diagnosis and received chemotherapy. We identified eight publications describing the use of CDV for adenovirus infection in the setting of HSCT or oncologic diagnoses treated with chemotherapy ( Table 3). Three of these studies ^14, 17, 27^ reported viral clearance in 89–100% of their patients. We observed similar rates of viral clearance (88%) but this was not consistently associated with clinical improvement.

There are very few reports on the use of CDV for adenovirus infection in pediatric SOT recipients, which have largely been restricted to reports of children receiving liver or lung transplants ^16, 20, 24, 25, 29, 30^. We identified six publications reporting the use of CDV for adenovirus infection in pediatric SOT recipients limited to one to four per report with the majority of these children having received liver or lung transplants ^16, 20, 24, 25, 29, 30^. Doan *et al.* ^25^ described children who had received lung transplants with reported viral clearance in three of their four patients. Our case series contributes patients who received several types of SOT including lung, heart, combined kidney and liver, and multi-visceral transplants. All patients with SOT in our series demonstrated viral clearance as well as resolution of symptoms, which may have reflected a combination of both the antiviral effect of CDV coupled with reduced immunosuppression. Interestingly, a prospective study performed in adult liver, kidney and heart transplant recipients noted the transient nature of adenoviremia and self limited infection in this population even in the absence of any treatment or interventions ^28^. However, the authors also note that the majority of patients in whom adenoviremia was detected in their study were asymptomatic or only mildly symptomatic, and thus differed from patients with severe infections who are more frequently reported in the pediatric transplant population and in lung allograft recipients. In contrast, Seidemann and colleagues ^29^ report mortality in 2 of 5 pediatric solid organ transplant recipients despite the use of cidofovir in their case series. Similarly, Leurez-Ville *et al.* ^30^ report mortality in 1 of 3 pediatric solid organ transplant recipients with disseminated adenovirus infection. The patients who expired in both these reports had severe symptoms and ultimately died of septic multiorgan failure. With the exception of a lung transplant recipient, all other solid organ transplant recipients with adenovirus infection in our study were more than mildly symptomatic. While other reports suggest the possibility of self-resolution of adenoviremia and absence of significant clinical sequelae in asymptomatic or mildly symptomatic solid organ transplant recipients, the propensity for serious infections and mortality has also been highlighted in the pediatric specific solid organ transplant literature ^29, 30^.

Two-thirds of our patients experienced resolution of their symptoms and had an overall favorable clinical course with recovery. However, one-third died all of whom were stem cell transplant recipients. With the exception of one patient it is unclear whether adenovirus was the direct cause of mortality in these patients. Our observations are consistent with what has been reported in the literature pertaining to outcomes in stem cell transplant recipients with adenovirus infections who have been treated with CDV ^9, 12, 14, 18, 23, 27^. Among SCT patients mortality remains high (10%–70%) even when clearance from blood is seen. It is hence difficult to conclude from these data whether or not cidofovir provided any clinical benefit. It should also be noted that two patients in our series received cidofovir despite being asymptomatic. One such patient was a lung transplant recipient in whom adenovirus was detected in both blood and BAL fluid, the other was a stem cell transplant recipient with a prior history of recurrent viremia and pneumonia. Both these patients were considered to be at high risk for complications from adenovirus infection thus leading to the decision to use cidofovir.

In our case series, renal dysfunction was common during CDV therapy with patients experiencing an average 50% increase of serum creatinine from their baseline. However, renal dysfunction was transient in the majority of patients with serum creatinine returning to baseline after cessation of CDV therapy. While some studies have reported no toxicities related to the use of CDV ^14, 16, 17, 19, 25^, the transient nature of nephrotoxicity observed has been reported by other studies ^20, 24^. Finally, brincidofovir (CMX001), an orally administered lipid -conjugate derivative of cidofovir is currently being investigated in clinical trials and is reported to have no nephrotoxicity ^31^.

Our study has several limitations. Most notably, the small number of patients precluded evaluation of other factors that may impact infection resolution such as immunosuppressive regimens, and additional factors that may impact degree of renal dysfunction. Nevertheless, our study adds to the limited reported literature of pediatric ADV patients treated with CDV.

## Data availability

The data referenced by this article are under copyright with the following copyright statement: Copyright: © 2016 Ganapathi L et al.

Data associated with the article are available under the terms of the Creative Commons Zero "No rights reserved" data waiver (CC0 1.0 Public domain dedication). 

*F1000Research*: Dataset 1. Raw data for Figure 1, 10.5256/f1000research.8374.d117321 ^32^

*F1000Research*: Dataset 2. Raw data for Figure 2, 10.5256/f1000research.8374.d117322 ^33^
