# Supplementary material for: Use of cidofovir in pediatric patients with adenovirus infection
Source: F1000Res. 2016 Dec 16;5:758. Originally published 2016 Apr 26. [Version 2] doi: 10.12688/f1000research.8374.2 (PMC4863673; doi:10.12688/f1000research.8374.2)
Supplement: Peer review history

# Peer review: Referee response for version 1

**DOI:** 10.5256/f1000research.9007.r13746

**Published:** 2016-05-09

**Authors:** David A Hunstad, Andrew Janowski

**Affiliations:** 1 Division of Infectious Diseases, Washington University in St Louis, St Louis, MO, USA; 2 Department of Pediatrics, Washington University in St Louis, St Louis, MO, USA

Ganapathi et al describe a case series of 16 patients who received a total of 19 courses of cidofovir for adenovirus infection. The authors provide virological data to demonstrate a response to cidofovir therapy, and provide additional data regarding renal function and co-administration of renal toxic medications. Ganapathi e *t al*. provides unique data regarding the peak creatinine level during therapy with cidofovir, though this value is difficult to interpret given the confounding factors of other nephrotoxic agents.

This case series is an incremental addition to the number of published cases of pediatric patients infected by adenoviruses who were treated with cidofovir. The manuscript follows a similar format to the case series reported by Bhadri *et al. *(2009), with presentation of the effects on renal function, and a summarization of the literature.

    

The authors should summarize outcomes from all other case series to provide updated overall mortality and nephrotoxicity of CDV-treated adenovirus infections in hematopoietic stem cell transplants, solid organ transplants, or other patients.  Such analyses may enhance the timeliness and utility of this manuscript.

In the discussion, the authors describe the potential benefit of cidofovir in solid organ transplant patients, in conjunction with reduced immunosuppression. However, without a comparative cohort of children who did not receive cidofovir or reduced immunosuppression, it would be difficult to draw this conclusion from the data presented. In addition, the authors do not discuss two interesting published papers. Humar *et al. *(2005) described a cohort of adult SOT patients with adenovirus viremia who did not receive cidofovir; all had spontaneous resolution of viremia with no deaths. In contrast, Seidemann *et al. *(2004) reported that 3 out of 5 pediatric SOT with adenoviremia (one also had a HSCT) died despite receiving cidofovir. The authors should absolutely cite these studies, especially Seidemann as it included pediatric SOT patients. In addition, the authors should highlight whether the positive outcomes in their SOT population are aligned with the adult study by Humar *et al. *(and the conclusion in that paper that treatment may not be necessary for adenoviremia in SOT patients); and contrast their results with those of Seidemann *et al.*

Specific comments

1. Methods: The authors should report their study site’s protocol for testing for adenoviruses, specifically, whether testing for adenoviruses was only performed on symptomatic patients, or if high-risk patients were routinely screened for adenovirus.
2. Methods: Probenecid is often co-administered with cidofovir. The authors should report if this drug was used in any of the courses of cidofovir, and what dosages. If probenecid was not used in all courses, the authors should analyze their data to investigate the role of probenecid in reducing nephrotoxicity.
3. Methods: It is not clear if adenovirus testing is completed on whole blood or serum. Later in the results, the authors state whole blood, but this should be explained in the methodology.
4. Methods: The authors should define pre-treatment/peak/post-treatment creatinine levels in their methodology. In addition, what defines pre-treatment creatinine? Is that the creatinine on the day of initiation of cidofovir? One week prior? Baseline from months prior? Defining this value is essential to the author’s statistical analysis.
5. Table 1: Patients should be ordered by diagnosis for better organization (as opposed to patient number); for example, the hematopoietic stem cell transplant patients should be grouped together, then the solid organ transplant patients should be grouped together. A column of outcomes should be added for greater clarity of which cases resulted in mortality, instead of the usage of an asterisk.
6. Results: Much of the information stated in the paragraph starting “Of the 19 courses prescribed” is redundant with the data presented in table 1, including the number of courses involving viremia, pneumonia, and GI symptoms. This paragraph can be consolidated, and the case characteristics presented as already implemented in Table 1.
7. Results: The authors should include a description of the patient’s response to cidofovir in Table 1. This would provide better organization and clarity in interpreting which characteristics were associated with response (or lack thereof) to therapy. It also would make the paragraph “We further examined the 16 blood-positive…” redundant, and this could be consolidated.
8. Figure 1: This figure might be easier to interpret if it were separated, for example, into two graphs of HSCT and SOT/other patients. It is difficult to follow 16 distinct virological lines in the current graph.  In addition, the color choices for patients 4, 9, and 13 are too similar.
9. The statistical analysis of increased risk of renal dysfunction when receiving 1 vs >2 renal toxic medications should be omitted as currently presented. The authors do not describe whether the additional nephrotoxic agents were co-administered at the same time or for what duration. These are critical confounding variables that are necessary for interpretation of whether nephrotoxicity was associated with increased administration of additional nephrotoxic agents. Given that this review is retrospective, such information may or may not be available. 
10. Figure 2 is a somewhat awkward way to describe SCr before and after CDV therapy.  In addition, it is unclear how, if only 64% of patients with elevated SCr returned to baseline, the mean SCr after CDV was equal to baseline (in the right bar of Figure 2). The table describes the SCr data adequately, and Figure 2 could therefore be omitted. 
11. The publication search criteria used to generate Table 3 should be explained in the methods. In their literature review, the authors should incorporate other pediatric adenovirus-cidofovir case reports and series that were not reviewed in this manuscript, including: Seidemann *et al. *(2004), Leruez-Ville *et al. *(2004), Nagafuji *et al. *(2004).
12. At least brief mention of the advent of brincidofovir for treatment of these patients with much reduced nephrotoxicity would be warranted.

We have read this submission. We believe that we have an appropriate level of expertise to confirm that it is of an acceptable scientific standard, however we have significant reservations, as outlined above.

## References

[1] BhadriVALee-HornLShawPJ: Safety and tolerability of cidofovir in high-risk pediatric patients. *Transpl Infect Dis*.2009;11(4) : 10.1111/j.1399-3062.2009.00391.x 373-9 10.1111/j.1399-3062.2009.00391.x 19392729

[2] HumarAKumarDMazzulliTRazonableRRMoussaGPayaCVCovingtonEAlecockEPescovitzMD: A surveillance study of adenovirus infection in adult solid organ transplant recipients. *Am J Transplant*.2005;5(10) : 10.1111/j.1600-6143.2005.01033.x 2555-9 10.1111/j.1600-6143.2005.01033.x 16162207

[3] SeidemannKHeimAPfisterEDKöditzHBeilkenASanderAMelterMSykoraKWSasseMWesselA: Monitoring of adenovirus infection in pediatric transplant recipients by quantitative PCR: report of six cases and review of the literature. *Am J Transplant*.2004;4(12) : 10.1111/j.1600-6143.2004.00631.x 2102-8 10.1111/j.1600-6143.2004.00631.x 15575915

[4] Leruez-VilleMMinardVLacailleFBuzynAAbachinEBlancheSFreymuthFRouziouxC: Real-time blood plasma polymerase chain reaction for management of disseminated adenovirus infection. *Clin Infect Dis*.2004;38(1) : 10.1086/380450 45-52 10.1086/380450 14679447

[5] NagafujiKAokiKHenzanHKatoKMiyamotoTEtoTNagatoshiYOhbaTObamaKGondoHHaradaM: Cidofovir for treating adenoviral hemorrhagic cystitis in hematopoietic stem cell transplant recipients. *Bone Marrow Transplant*.2004;34(10) : 10.1038/sj.bmt.1704682 909-14 10.1038/sj.bmt.1704682 15361907

# Response

**Published:** 2016-12-05

**Authors:** Lakshmi Ganapathi

**Affiliations:** Boston Children's Hospital, USA

Addressing Dr Hunstad’s and Dr Janowski’s report, in the new version of the manuscript we have clarified the following in the methods section: 1) The protocol for testing for adenovirus infection, 2) Usage of probenecid and dose (it was used in all patients who received cidofovir), 3) how we defined pre-treatment creatinine and 4) the publication search criteria. While we mention the use of concomitant medications that are known to render nephrotoxicity, we agree that the analysis is confounded by several factors and hence have omitted the statistical analysis of increased risk of renal dysfunction when receiving 1 versus >1 nephrotoxic medications.

 In the discussion section, we have expanded our discussion on the potential benefit of cidofovir in solid organ transplant recipients, and compare the findings of Humar et al  (a study in which patients were all adults) with the findings reported by Seidemann et al and Leruez-Ville et al. As such, we added the latter two studies to table 3 for an updated literature review. However, we did opt to leave out the study by Nagafuji et al as recommended by the reviewers. The study by Nagafuji et al  focused primarily on adult HSCT recipients and included only one pediatric patient. We feel that given much larger pediatric specific case series of HSCT recipients with adenovirus infection, this one case reported adds little. On the other hand, we did include case reports in the solid-organ patient literature review given the paucity of data in this population. We have also made a brief mention regarding the advent of brincidofovir.

 We updated table 1 to include include a column of outcomes. While we recognize that other changes as recommended by the reviewers may contribute to better organization of text and data, we did not make too many other changes to table 1 or figure 1 given that these changes are stylistic and not necessarily content relevant. 

# Peer review: Referee response for version 1

**DOI:** 10.5256/f1000research.9007.r13587

**Published:** 2016-05-04

**Authors:** Jeffrey Bergelson

**Affiliations:** 1 Department of Pediatrics, University of Pennsylvania School of Medicine, Philadelphia, PA, USA

This paper reports the use of cidofovir for treatment of adenovirus infection in 16 pediatric patients, and provides detailed information about the patients’ clinical status, blood viral loads, and renal function throughout the treatment course.

I have only one major point for the authors to consider. Most patients survived, and a number had decreases in viral load within several weeks of the start of cidofovir therapy. However, the patient group was quite heterogeneous (one case had cerebral palsy, but no apparent immune dysfunction); in some cases the viral loads rose for a long time before they began to fall; and 4 of the 7 patients who had undergone allogeneic stem cell transplant died. I think the discussion should point out explicitly that it is difficult to conclude from these data whether or not cidofovir provided any clinical benefit.

Minor points:

 

1. Clarify the assay used for viral loads. Is it the Argene assay, or an in-house assay using some components of the Argene kit?
2. Clarify the definition of “viral response”. Is it a 10-fold decrease in titer?
3. Perhaps mention briefly why the asymptomatic patients were treated with cidofovir. It appears that one was a lung transplant patient (considered to be at high risk) and one a SCT patient with recurrent viremia.
4. Figure 2 adds little.

Typos, spelling, glitches:

Page 3.  “These associated adverse effects”;  THIS adverse EFFECT.

Page 3.   “1 mg/kg three times” WEEKLY

Page 3.   “Respiratory tract”; italicize

Page 3.   “The peak SCr during therapy was used to calculate the number…” should be deleted.

Table 1.  “multivisCeral”

Page 5.   “Respiratory symptoms were the most common presentation in 10 courses”; should be “… most common presentation (10 courses)..”

Page 5.  “Two courses were prescribed… respectively” ; should be One course was prescribed in asymjptomatic x, and one in y.

Page 5.  “improvement with viral clearance, however”; comma should be a semicolon.

Page 6.  “.. one third died all of which”; ?  …died, all of WHOM..”

I have read this submission. I believe that I have an appropriate level of expertise to confirm that it is of an acceptable scientific standard.

# Response

**Published:** 2016-05-05

**Authors:** Jeffrey Bergelson

**Affiliations:** Department of Pediatrics, University of Pennsylvania School of Medicine, USA

Because the paper provides no evidence about efficacy, the abstract should be changed, as well as the discussion. "….was associated with viral response and clinical improvement" should be softened.

# Response

**Published:** 2016-12-05

**Authors:** Lakshmi Ganapathi

**Affiliations:** Boston Children's Hospital, USA

Addressing Dr Bergelson’s report, in the new version of the manuscript we have expanded our discussion to point out more explicitly that at least based on our data, while we did observe clearance of viremia in several patients overall clinical benefit was somewhat harder to conclude. We have also mentioned the rationale behind treating asymptomatic patients (both treated patients were considered to be at high risk for complications).

 In the methods section, we have clarified the following: 1) The assay used to determine viral load, 2) What we defined as viral response.

 We have also corrected the minor spelling and punctuation issues.

 We agree with both  Dr Bergelson and Dr Hundstad that Figure 2 adds little and have removed it from this version of the manuscript

# Peer review: Referee response for version 1

**DOI:** 10.5256/f1000research.9007.r13586

**Published:** 2016-05-03

**Authors:** Miguel O’Ryan

**Affiliations:** 1 Institute of Biomedical Sciences, Faculty of Medicine, Universidad de Chile, Santiago, Chile

This is a well written descriptive, retrospective study of a case series of immunocompromised children receiving cidofovir for treatment of mostly probable, few definite or asymptomatic adenovirus infections. Children had different underlying diseases including a few with solid organ transplantation. The series review is transparent, showing viral and clinical evolution as well as renal compromise in association with treatment. Most children cleared adenovirus with treatment but as the authors point out, also in association with improved immunity. Most but not all children clearing the virus improved clinically, a few died despite clearing the virus. Transient nephrotoxicity occurred in near 50% of children, but this occurred in association with other nephrotoxic treatments and was not a major problem. This review adds to the rather limited number of currently available series and although not providing any definite conclusion (clinicians will still have to balance several variables before deciding to use cidofovir or not) it adds helpful information for treating physicians that have to make these hard decisions.

I have read this submission. I believe that I have an appropriate level of expertise to confirm that it is of an acceptable scientific standard.
